# Does weight matter in cryoballoon ablation?

**DOI:** 10.1016/j.ijcha.2023.101251

**Published:** 2023-07-26

**Authors:** Monika Gawałko, Dominik Linz, Dobromir Dobrev

**Affiliations:** 1st Department of Cardiology, Medical University of Warsaw, Warsaw, Poland; Institute of Pharmacology, West German Heart and Vascular Center, University Duisburg-Essen, Germany; Department of Cardiology, Maastricht University Medical Centre and Cardiovascular Research Institute Maastricht, Maastricht, the Netherlands; Department of Cardiology, Maastricht University Medical Centre and Cardiovascular Research Institute Maastricht, Maastricht, the Netherlands; Department of Biomedical Sciences, Faculty of Health and Medical Sciences, University of Copenhagen, Copenhagen, Denmark; Centre for Heart Rhythm Disorders, Royal Adelaide Hospital, University of Adelaide, Adelaide, Australia; Institute of Pharmacology, West German Heart and Vascular Center, University Duisburg-Essen, Germany; Department of Integrative Physiology, Baylor College of Medicine, Houston, USA; Department of Medicine and Research Center, Montréal Heart Institute and Université de Montréal, Montréal, Canada

Atrial fibrillation (AF) is the most common cardiac arrhythmia and is associated with an increased risk of thromboembolic events, heart failure, mortality, and impaired quality of life [1], [2]. Despite the growing knowledge about the underlying molecular mechanisms of AF [3], therapeutic approaches remain suboptimal. Catheter-based pulmonary vein isolation represents the cornerstone of modern heart rhythm control strategies. Cryoballoon ablation (CBA) is a procedure commonly used to treat AF [4], [5] with comparable efficacy and safety to radiofrequency ablation in patients with paroxysmal AF [6], while its value in persistent AF is less clear and is under investigation in an randomized trial [7]. Regardless of energy source, it is known that AF catheter ablation success depends on various risk factors, such as obesity and diabetes, which may cause silent brain infarction [8], failure to restore sinus rhythm [9] and AF-associated adverse outcomes [10]. Although the impact of obesity on procedure complications [11] and outcomes [12] is well established for radiofrequency ablation, there is limited information available regarding CBA.

When CBA is performed in obese patients, several considerations need to be taken into account. Obese patients tend to have thicker chest walls and increased amounts of fat tissue, which may make catheter movements and adequate visualization of the pulmonary vein occlusion challenging despite increased radiation exposure for the patient and the operator during the CBA procedure. Besides this, obese patients may have a higher risk of anesthesia-related complications due to comorbidities such as obstructive sleep apnea and respiratory issues [13], [14], [15]. It is generally accepted that CBA procedures may take longer in obese patients compared to non-obese patients, although efficacy and safety of CBA in obese patients appear to be comparable to non-obese patients [16], [17].

The present study by Boehmer et al. [18] shows that CBA may be equally effective in reducing AF recurrences in obese as compared to non-obese patients, with no worsening of safety endpoints. In a group of 949 participants with a median 15-month follow-up, AF recurrences occurred in 30% (76/251) of obese and 35% (243/698) of non-obese patients (p < 0.001 for non-inferiority). However, procedural parameters including the procedure time and radiation dose were significantly higher in obese vs non-obese patients. In a subgroup analysis, the occurrence of recurrent AF was unaffected by the presence of diabetes.

Many issues need consideration when interpreting the present results. The categorisation of patients into obese vs non-obese resulted in a small difference in body mass index (BMI) with partially overlapping values between both groups. Only a small proportion (23%) of patients had a BMI ≥ 35 kg/m^2^ in the obese group. At the same time, more than half (55%) of all patients in the non-obese group had overweight (BMI between 25 and 30 kg/m^2^). BMI is a continuous variable and splitting patient into two groups based on a certain threshold is clinically overly simplistic as being severely overweight with a BMI of 29.9 kg/m^2^ is unlikely to be associated with a different biological risk than being obese with a BMI of 30 kg/m^2^.

Interestingly, older age and persistent AF were independent predictors of recurrent AF. Although no difference in occurrence of persistent AF was observed between both groups, patients in the non-obese (vs obese) group were significantly older. This may represent the manifestation of a selection bias, where older (chronologically and biologically) and therefore more fragile obese patients were not scheduled for catheter ablation but managed differently with antiarrhythmic drugs or rate control strategies. Additionally, BMI is not a precise readout of the obesity-AF interaction. Older patients have increased ectopic fat accumulation in seemingly lean or slightly overweight patients, which increases the likelihood to be included in the non-obese patient group [19]. Advanced age is associated with a shift in fat distribution to visceral adipose tissue, along with a reduction in subcutaneous adipose tissue mass. As the visceral fat is unevenly distributed throughout the body, people may age with a localization of fat tissue around the internal organs including heart [20] and appear visually skinny, or with abdominal location of visceral tissue and demonstrate obese silhouette. This might have partially averaged out the difference between the obese and non-obese AF group concerning the AF outcome after CBA.

Another factor that needs consideration is the mode of assessment of AF recurrences. The follow-up involved heart rhythm assessments using either a 12-lead or 1-lead electrocardiogram at 6-month intervals over a period of 48 months, or electrocardiogram evaluation in response to reported symptoms during telephone interviews. Consequently, it might potentially result in missed episodes of AF. Nevertheless, it is important to note that this potential limitation applies to both non-obese and obese patients.

In predefined subgroup analysis, the authors found that the presence of diabetes does not seem to influence the efficacy of CBA. These results do not confirm previous findings from the literature. As stressed by the authors [18], one possible explanation for the inconsistent results could be a good glycaemic control of the study participants (HbA1c 6.6% in non-obese and 6.9% in obese patients). This is consistent with previous findings showing that HbA1c levels below 7.0% are associated with low AF recurrence rates [21].

In summary, although AF recurrence rate appears to be not affected by body weight future controlled studies are required to validate the notion that the independence of AF recurrence rate from body weight specifically applies to CBA. Until then, patients scheduled for AF ablation should be advised to lose weight and to work on their overall cardiovascular risk profile to prevent future cardiovascular complications. Finally, longer procedure times and higher radiation exposure that complicate elective CBA procedures in obese patients can be avoided by pre-procedural weight loss interventions.

## Sources of Funding

The authors research is supported by National Institutes of Health (R01-HL131517, R01-HL136389, R01-HL089598, R01HL163277, and R01HL160992 to D.D.), and European Union (large-scale integrative project MAESTRIA, No. 965286 to D.D).

## Declaration of Competing Interest

The authors declare that they have no known competing financial interests or personal relationships that could have appeared to influence the work reported in this paper.
